# DeepGreen: deep learning of Green’s functions for nonlinear boundary value problems

**DOI:** 10.1038/s41598-021-00773-x

**Published:** 2021-11-03

**Authors:** Craig R. Gin, Daniel E. Shea, Steven L. Brunton, J. Nathan Kutz

**Affiliations:** 1 Department of Population Health and Pathobiology, North Carolina State University, Raleigh, NC 27695 USA; 2 Department of Materials Science and Engineering, University of Washington, Seattle, WA 98195 USA; 3 Department of Mechanical Engineering, University of Washington, Seattle, WA 98195 USA; 4 Department of Applied Mathematics, University of Washington, Seattle, WA 98195 USA

**Keywords:** Applied mathematics, Computational science, Engineering, Mathematics and computing, Software

## Abstract

Boundary value problems (BVPs) play a central role in the mathematical analysis of constrained physical systems subjected to external forces. Consequently, BVPs frequently emerge in nearly every engineering discipline and span problem domains including fluid mechanics, electromagnetics, quantum mechanics, and elasticity. The fundamental solution, or Green’s function, is a leading method for solving linear BVPs that enables facile computation of new solutions to systems under any external forcing. However, fundamental Green’s function solutions for nonlinear BVPs are not feasible since linear superposition no longer holds. In this work, we propose a flexible deep learning approach to solve nonlinear BVPs using a dual-autoencoder architecture. The autoencoders discover an invertible coordinate transform that linearizes the nonlinear BVP and identifies both a linear operator *L* and Green’s function *G* which can be used to solve new nonlinear BVPs. We find that the method succeeds on a variety of nonlinear systems including nonlinear Helmholtz and Sturm–Liouville problems, nonlinear elasticity, and a 2D nonlinear Poisson equation and can solve nonlinear BVPs at orders of magnitude faster than traditional methods without the need for an initial guess. The method merges the strengths of the universal approximation capabilities of deep learning with the physics knowledge of Green’s functions to yield a flexible tool for identifying fundamental solutions to a variety of nonlinear systems.

## Introduction

Boundary value problems (BVPs) are ubiquitous in the sciences^1^. From elasticity to quantum electronics, BVPs have been fundamental in the development and engineering design of numerous transformative technologies of the 20th century. Historically, the formulation of many canonical problems in physics and engineering result in *linear* BVPs: from Fourier formulating the heat equation in 1822^2^ to more modern applications such as designing chip architectures in the semi-conductor industry^3,4^. Much of our theoretical understanding of BVPs comes from the construction of the fundamental solution of the BVP, commonly known as the Green’s function^5^. The Green’s function solution relies on a common property of many BVPs: *linearity*. Specifically, general solutions rely on linear superposition to hold, thus limiting their usefulness in many modern applications where BVPs are often heterogeneous and nonlinear. By leveraging modern deep learning, we are able to learn linearizing transformations of BVPs that render *nonlinear BVPs linear* so that we can construct the Green’s function solution. Our deep learning of Green’s functions, *DeepGreen*, provides a transformative architecture for modern solutions of nonlinear BVPs.

DeepGreen is inspired by recent works which use deep neural networks (DNNs) to discover advantageous coordinate transformations for dynamical systems^6–15^. The universal approximation properties of DNNs^16,17^ are ideal for learning coordinate transformations that linearize nonlinear BVPs, ODEs and PDEs. Specifically, such linearizing transforms fall broadly under the umbrella of Koopman operator theory^18^, which has a modern interpretation in terms of dynamical systems theory^19–22^. There are only limited cases in which Koopman operators can be constructed explicitly^23^. However *Dynamic Mode Decomposition* (DMD)^24^ provides a numerical algorithm for approximating the Koopman operator^25^, with many recent extensions that improve on the DMD approximation^26^. More recently, neural networks have been used to construct Koopman embeddings^6,8–13,15^. This is an alternative to enriching the observables of DMD^27–33^. Thus, neural networks have emerged as a highly effective mathematical tool for approximating complex data^34,35^ with a *linear* model. DNNs have been used in this context to discover time-stepping algorithms for complex systems^36–40^. Moreover, DNNs have been used to approximate constitutive models of BVPs^41^. Recent works have used neural network architectures for identifying operators of BVPs^42^ and learning manifolds^43^ on which high-dimensional PDEs can be solved. However, these works fail to guarantee discovery of an invertible operator or linearize nonlinear systems.

**Figure 1:**
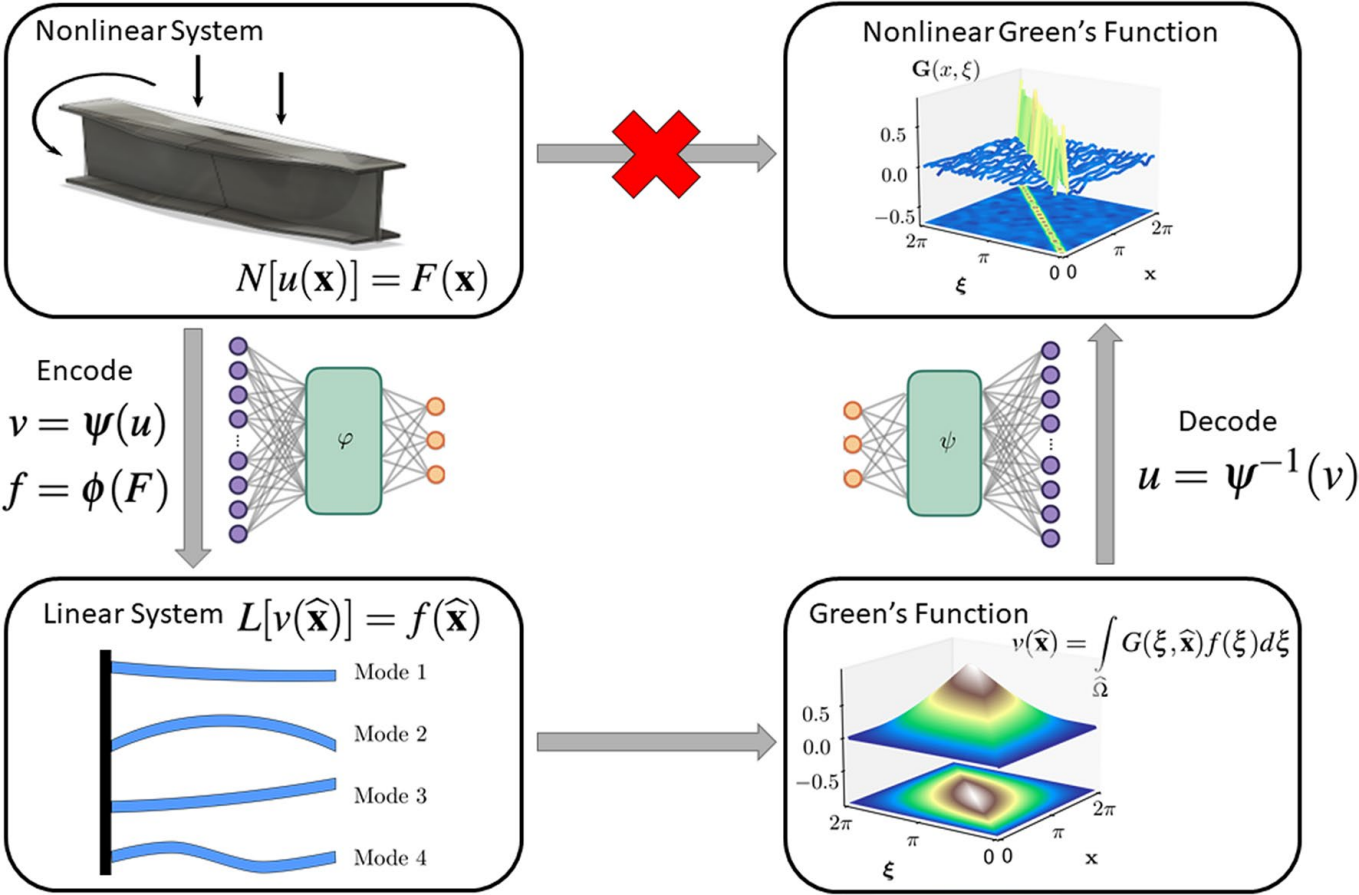
DeepGreen solves nonlinear BVPs by identifying the Green’s Function of the nonlinear problem using a deep learning approach with a dual autoencoder architecture. A nonhomogenous linear BVP can be solved using the Green’s function approach, but a nonlinear BVP cannot. DeepGreen transforms a nonlinear BVP to a linear BVP, solves the linearized BVP, and then inverse transforms the linear solution to solve the nonlinear BVP.

DeepGreen leverages the success of DNNs for dynamical systems to discover coordinate transformations that linearize nonlinear BVPs so that the Green’s function solution can be recovered. This allows for the discovery of the fundamental solutions for nonlinear BVPs, opening many opportunities for the engineering and physical sciences. DeepGreen exploits physics-informed learning by using autoenconders (AEs) to take data from the original high-dimensional input space to the new coordinates at the intrinsic rank of the underlying physics^6,7,44^. The architecture also leverages the success of *Deep Residual Networks* (DRN)^45^ which enables our approach to efficiently handle near-identity coordinate transformations^15^ by borrowing the concept of skip connections. Figure 1 highlights the deep learning approach which leverages a dual autoencoder architecture. DeepGreen transforms a nonlinear BVP to a linear BVP, solves the linearized BVP, and then inverse transforms the linear solution to solve the nonlinear BVP.

The Green’s function constructs the solution to a BVP for any given forcing by superposition. Specifically, consider the classical linear BVP^5^

1 $$\begin{aligned} \begin{array}{ll} L[v({{\mathbf {x}}})] = f({{\mathbf {x}}}), \end{array} \end{aligned}$$

where *L* is a linear differential operator, *f* is a forcing, ${{\mathbf {x}}}\in {\Omega }$ is the spatial coordinate, and ${\Omega }$ is an open set. The boundary conditions $Bv({{\mathbf {x}}})=0$ are imposed on $\partial {\Omega }$ with a linear operator *B*. The fundamental solution is constructed by considering the adjoint equation

2 $$\begin{aligned} \begin{array}{ll} L^\dag [G({{\mathbf {x}},{\xi }})] = \delta ({{\mathbf {x}}}-{{\xi }}), \end{array} \end{aligned}$$

where $L^\dag$ is the adjoint operator (along with its associated boundary conditions) and $\delta ({{\mathbf {x}}}-{{\xi }})$ is the Dirac delta function. Taking the inner product of (1) with respect to the Green’s function gives the fundamental solution

3 $$\begin{aligned} v(\mathbf{x}) = (f({{\xi }}), G({\xi },\mathbf{x}) ) = \int \limits _{{\Omega }} G(\mathbf {\xi },\mathbf{x}) f({\xi }) d{\xi }, \end{aligned}$$

which is valid for any forcing $f({{\mathbf {x}}})$. Thus once the Green’s function is computed, the solution for arbitrary forcing functions can be easily extracted from integration. This integration represents a superposition of a continuum of delta function forcings that are used to represent $f({{\mathbf {x}}})$.

There have been many recent works on learning kernel representations of operators^46–49^ including several that use deep learning algorithms^50–53^. The representation of the solution as an integration over a kernel function (3) has been recently exploited using deep learning algorithms^50–53^. Indeed, a representation of the Green’s function kernel is explicitly learned in Li et al.^51^ for linear, non-constant coefficent PDEs. A critical difference here is that we consider nonlinear BVPs for which one must learn a coordinate transformation and kernel representation jointly. This is a significant difference in outlook as we can turn nonlinear problems linear so as to exploit linear superposition. Indeed, in many modern applications, nonlinearity plays a fundamental role so that the BVP is of the form

4 $$\begin{aligned} \begin{array}{ll} N[u({{\mathbf {x}}})] = F({{\mathbf {x}}}), \end{array} \end{aligned}$$

where *N* is a nonlinear differential operator. For this case, the principle of linear superposition no longer holds and the notion of a fundamental solution is lost. However, modern deep learning algorithms allow us the flexibility of learning coordinate transformations (and their inverses) of the form

5 $$\begin{aligned} v&= \varvec{\psi }(u), \end{aligned}$$

6 $$\begin{aligned} f&= \varvec{\phi }(F), \end{aligned}$$

such that *v* and *f* satisfy the linear BVP (1) for which we generated the fundamental solution (3). This gives a nonlinear fundamental solution through use of this deep learning transformation.

DeepGreen is a *supervised learning* algorithm which is ultimately a high-dimensional interpolation problem^54^ for learning the coordinate transformations $\varvec{\psi }(u)$ and $\varvec{\phi }(F)$. DeepGreen is enabled by a physics-informed deep autoencoder coordinate transformation which establishes superposition for nonlinear BVPs, thus enabling a Koopman BVP framework. The learned Green’s function enables accurate construction of solutions with new forcing functions in the same way as a linear BVP, thus enabling the solution of nonlinear BVPs in a fraction of the time it takes to solve them using traditional solvers and without the need for an initial guess. We demonstrate the DeepGreen method on a variety of nonlinear boundary value problems, including a nonlinear 2D Poisson problem, showing that such an architecture can be used in many modern and diverse applications in aerospace, electromagnetics, elasticity, materials, and chemical reactors.

## Results

**Figure 2:**
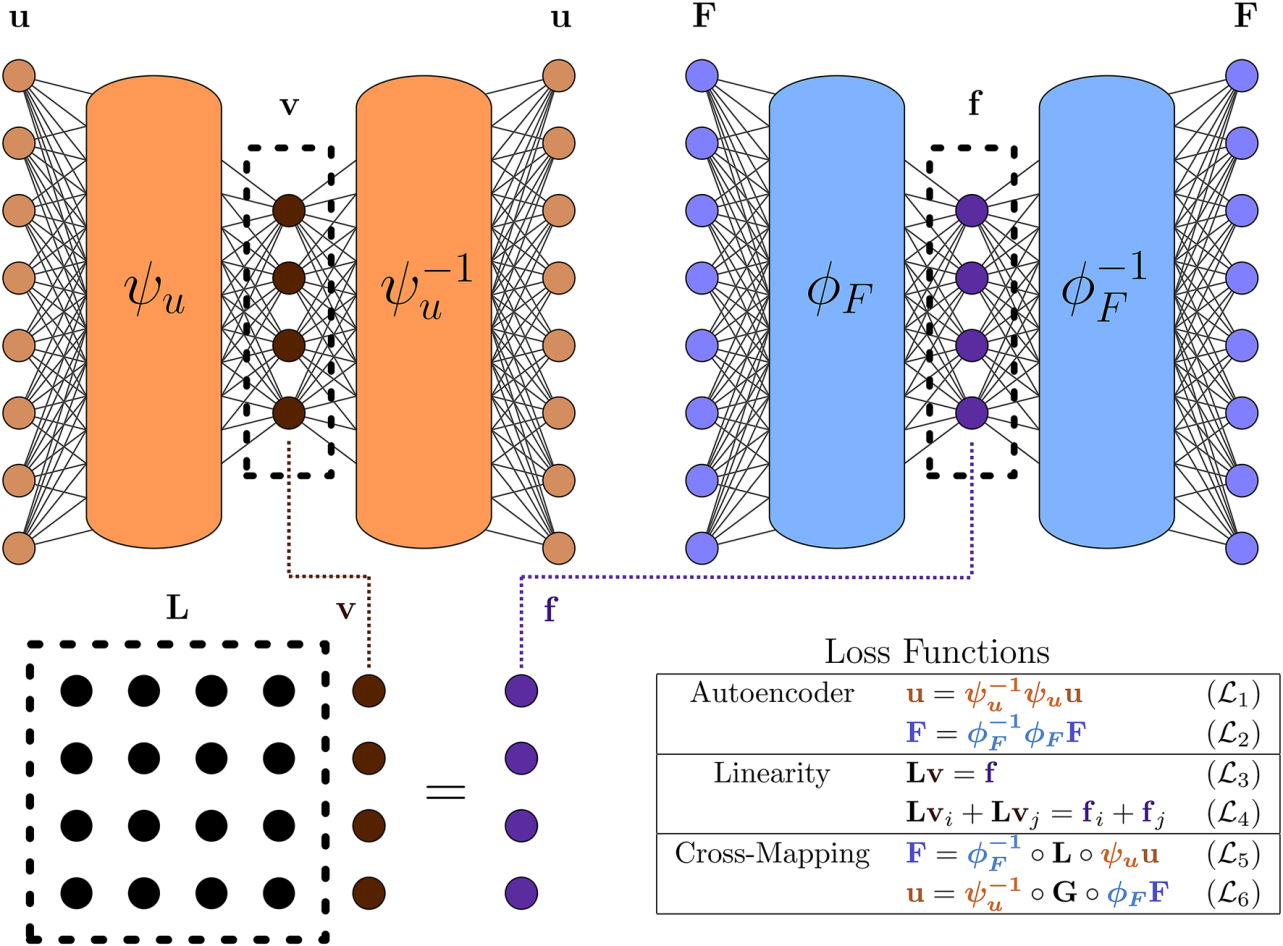
DeepGreen architecture. Two autoencoders learn invertible coordinate transformations that linearize a nonlinear boundary value problem. The latent space is constrained to exhibit properties of a linear system, including linear superposition, which enables discovery of a Green’s function for nonlinear boundary value problems.

The DeepGreen architecture, which features two autoencoders to learn invertible coordinate transformations that linearize a nonlinear boundary value problem, is highlighted in Fig. 2. The associated loss functions are discussed in the “Methods: deep autoencoders for linearizing BVPs” section. Here we demonstrate its success on a number of canonical nonlinear BVPs. The first three BVPs are one-dimensional systems and the final one is a two-dimensional system. The nonlinearities in these problems do not allow for a fundamental solution, thus recourse is typically made to numerical computations to achieve a solution. DeepGreen, however, can produce a fundamental solution which can then be used for any new forcing of the BVP.

**Figure 3:**
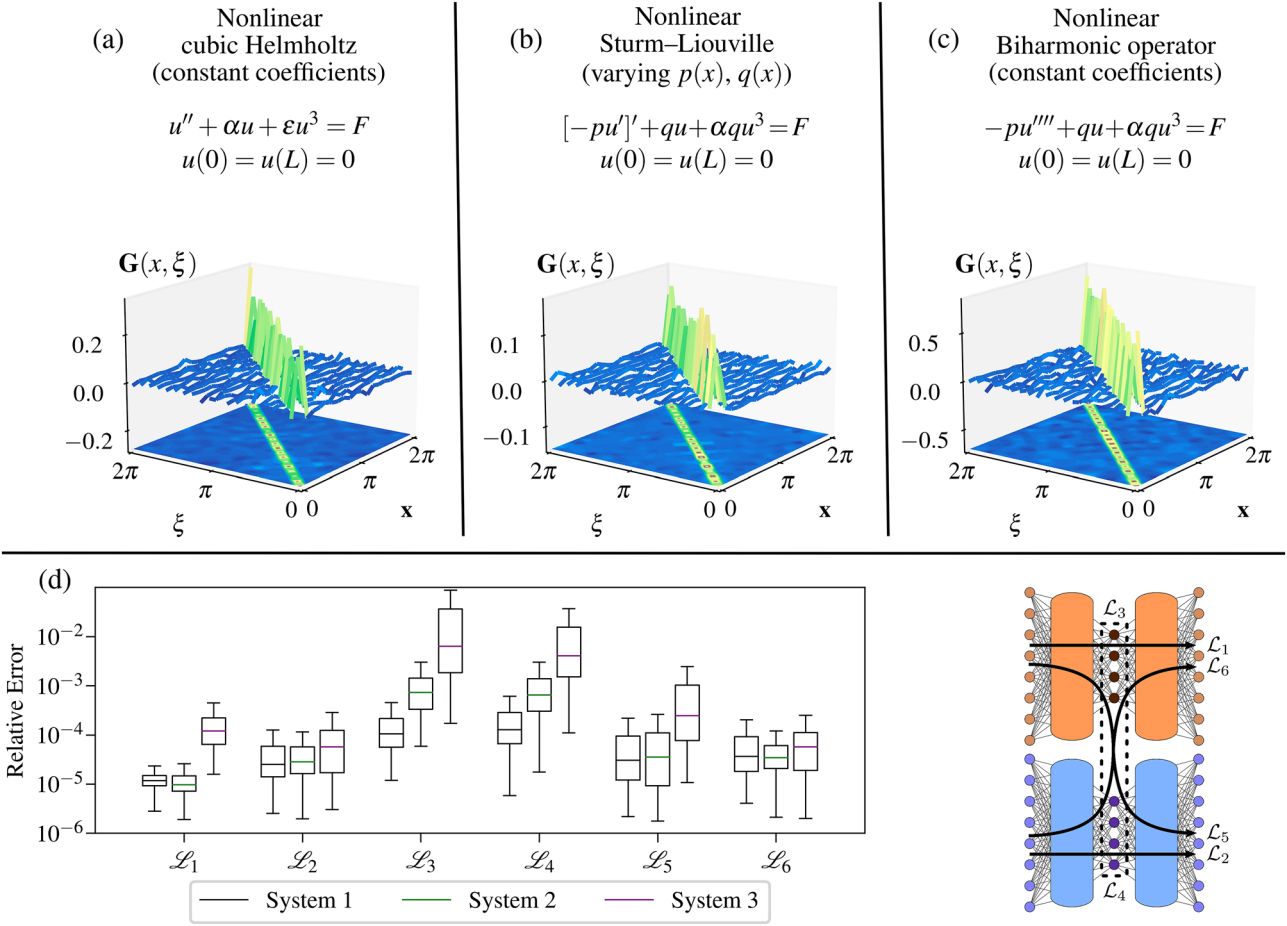
Summary of results for three one-dimensional models. The models and the Green’s function learned by DeepGreen are given for (**a**) a nonlinear Helmholtz equation, (**b**) a nonlinear Sturm–Liouville equation, and (**c**) a nonlinear biharmonic operator. (**d**) A summary box plot shows the relative losses ${\mathcal {L}}_1$, ${\mathcal {L}}_2$, ${\mathcal {L}}_3$, ${\mathcal {L}}_4$, ${\mathcal {L}}_5$, and ${\mathcal {L}}_6$ for all three model 1D systems.

### One-dimensional examples

We first applied the DeepGreen methodology to three different one-dimensional BVPs. The first problem is a nonhomogeneous second-order nonlinear Sturm–Liouville model with constant coefficients and a cubic nonlinearity, thus making it a cubic Helmholtz equation. The differential equation is given by

7 $$u^{\prime\prime} + \alpha u + \varepsilon u^3 = F(x),$$

8 $$\begin{aligned}u(0) = u(2\pi ) = 0, \end{aligned}$$

where $u=u(x)$ is the solution when the system is forced with *F*(*x*) with $x \in [0,2\pi ]$, $\alpha = -1$ and $\varepsilon = - 0.3$. The notation $u''$ denotes $\frac{d^2}{dx^2}u(x)$. The second system is governed by the nonlinear Sturm–Liouville equation

$$\begin{aligned}&[-p(x) u^{\prime}]^{\prime} + q(x) (u + \varepsilon u^3) = F(x), \\& u(0) = u(2\pi ) = 0, \end{aligned}$$

where $\varepsilon = 0.4$ controls the extent of nonlinearity, and *p*(*x*) and *q*(*x*) are spatially-varying coefficients

$$\begin{aligned} p(x)= & 0.5 \sin (x) - 3, \\ q(x)= & 0.6 \sin (x) - 2 , \end{aligned}$$

with $x \in [0,2\pi ]$. The final one-dimensional system is a biharmonic operator with an added cubic nonlinearity

$$\begin{aligned} & [-p u^{\prime\prime}]^{\prime\prime} + q (u + \varepsilon u^3) = F(x), \\&u(0) = u(2\pi ) = u'(0) = u'(2\pi ) = 0, \end{aligned}$$

where $p=-4$ and $q=2$ are the coefficients and $\varepsilon =0.4$ controls the nonlinearity.

Each dataset contains discretized solutions and forcings, $\{{\mathbf {u}}_k,{\mathbf {F}}_k\}_{k=1}^N$. The forcing functions used for training are cosine and Gaussian functions; details of data generation and the forcing functions are provided in the Supplementary Materials. The data is divided into three groups: training, validation, and test. The training and validation sets are used for training the model. The test set is used to evaluate the results. The training set contains $N_{train}=8906$ vector pairs ${\mathbf {u}}_k$ and ${\mathbf {F}}_k$. The validation set contains $N_{validation}=2227$ pairs, and the test set contains $N_{test}=1238$. Training and performance evaluation are discussed in the “Methods: deep autoencoders for linearizing BVPs” section.

Results for all the one-dimensional models are presented in Fig. 3. The model performance is quantitatively summarized by box plots and the Green’s function matrix is shown for each model. The results of Fig. 3 demonstrate that the DeepGreen architecture enables the discovery of invertible, linearizing transformations that facilitate identification of a linear operator and Green’s function to solve nonlinear BVPs. Importantly, the learned operators and Green’s function matrices consistently exhibit a diagonally-dominant structure, which hints at the model’s preference to learn an optimal basis. The losses for the nonlinear cubic Helmholtz equation and the nonlinear Sturm–Liouville equation are similar which indicates that spatially-varying coefficients do not make the problem significantly more difficult for the DeepGreen architecture. In contrast, the losses for the nonlinear biharmonic equation are about an order of magnitude higher than the other two systems. This result implies the fourth-order problem is more difficult than the second-order problems. The linear operator loss ${\mathcal {L}}_3$ and superposition loss ${\mathcal {L}}_4$ are consistently the highest losses across all models. This indicates that DeepGreen easily identifies effective invertible autoencoding schemes and incurs most of its error from the discovered operator. This dynamic emphasizes the importance of finding an optimal operator during training that works well with the simultaneously discovered autoencoder transform.

Serving as an example, the cubic Helmholtz model is tested on data similar and dissimilar to the training data, and evaluated on the loss functions that guide the training procedure (see Fig. 6 in the “Methods: deep autoencoders for linearizing BVPs” section). The model appears to extrapolate beyond the test data, suggesting that the learned operator is somewhat general to the system.

### Nonlinear Poisson equation

We also tested our method on a two-dimensional system. The two-dimensional model is a nonlinear version of the Poisson equation with Dirichlet boundary conditions

9 $$-\nabla \cdot \left[ (1+u^2) \nabla u\right] = F({\mathbf {x}}),\quad {\mathbf {x}}\in \Omega , $$

10 $$u = 0, \quad {\mathbf {x}}\in \partial \Omega,$$

where $\Omega := (0,2\pi ) \times (0,2 \pi )$. Similar to the one-dimensional models, the forcing functions used to train the model are cosine and Gaussian functions, the details of which are provided in the Supplementary Materials. The sizes of the data sets are also similar to the one-dimensional data sets. The training data contains $N_{train}=9806$ vector pairs ${\mathbf {u}}_k$ and ${\mathbf {F}}_k$, the validation data contains $N_{validation}=2452$, and the test data contains $N_{test}=1363$.

**Figure 4:**
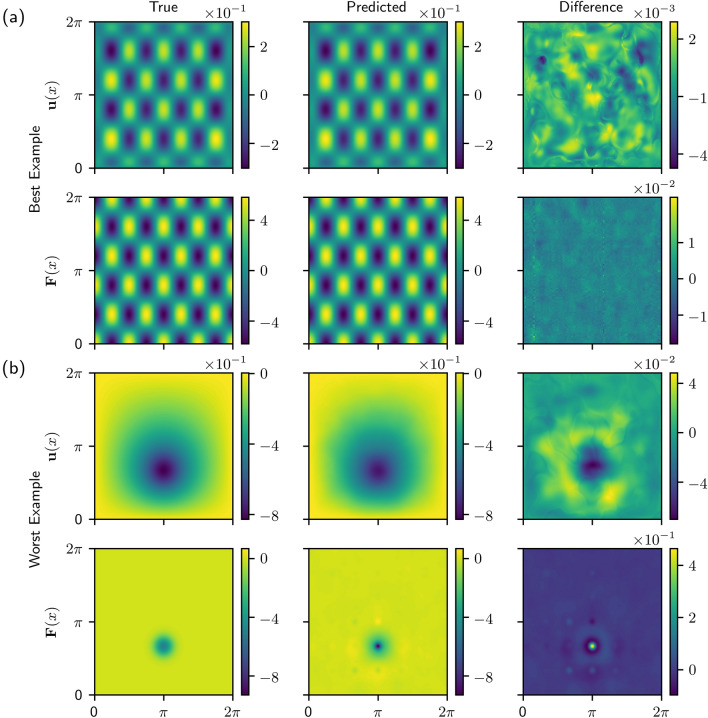
Model predictions for the (**a**) best and (**b**) worst examples from test data with Gaussian and cosine forcings. In both (**a,b**), the top row shows the true solution ${\mathbf {u}}({\mathbf {x}})$, the predicted solution using the Green’s function, and the difference between the true and predicted solution. The bottom row shows the true forcing function ${\mathbf {F}}({\mathbf {x}})$, the predicted forcing function, and the difference between the true and predicted forces. In order to account for the difference in scale between ${\mathbf {u}}({\mathbf {x}})$ and ${\mathbf {F}}({\mathbf {x}})$, the differences are scaled by the infinity norm of the true solution or forcing function ($\text {Difference} = (\text {True} - \text {Predicted})/ || \text {True}||_{\infty }$).

The model was evaluated on test data containing cosine and Gaussian forcing functions. Figure 4a shows the true solution ${\mathbf {u}}(x)$ and forcing function ${\mathbf {F}}(x)$ as well as the network predictions for the example from the test data for which the model performed the best (i.e. the smallest value of the loss). The difference between the true and predicted functions is shown in the right column of Fig. 4a and is scaled by the infinity norm of the true solution or forcing functions. Figure 4b shows similar results but for the worst example from the test data. In both cases, the model gives a qualitatively correct solution for both ${\mathbf {u}}(x)$ and ${\mathbf {F}}(x)$. Unsurprisingly, the network struggles most on highly localized forcing functions and has the highest error in the region where the forcing occurs.

## Discussion

We have leveraged the expressive capabilities of deep learning to discover linearizing coordinates for nonlinear BVPs, thus allowing for the construction of the *fundamental solution or nonlinear Green’s function*. Our architecture leverages two autoencoders to simultaneously learn coordinates and operators for expressing the solution in its kernel (Green’s function) representation. Much like the Koopman operator for time-dependent problems, the linearizing transformation provides a framework whereby the fundamental solution of the linear operator can be constructed and used for any arbitrary forcing. This provides a broadly applicable mathematical architecture for constructing solutions for nonlinear BVPs, which typically rely on numerical methods to achieve solutions. Our DeepGreen architecture can achieve solutions for arbitrary forcings by simply computing the convolution of the forcing with the Green’s function in the linearized coordinates.

Because solving in the linearized coordinates is so simple, the trained neural networks allow for significant speed advantages over traditional methods. In particular, we found that solving the one-dimensional systems with a traditional solver takes over 10,000 times longer than solving with DeepGreen. For the cubic Helmholtz equation, the speed up from using DeepGreen was over 50,000 times. For the two-dimensional system, the traditional method considered takes 126 times longer than DeepGreen which still provides significant advantages for applications. More details on the speed test can be found in the Supplementary Materials. Another advantage of DeepGreen is that unlike many traditional BVP solvers, it does not require an initial guess and therefore its success is not dependent on obtaining a good enough initialization for convergence. In addition to providing a means to solving BVPs, DeepGreen gives access to an analogue of the Green’s function for nonlinear BVPs. Green’s functions give insight into properties of the BVP and the underlying physical system^5^ and can be used to devise fast and efficient numerical algorithms^55–60^. Therefore, the DeepGreen architecture opens the door to techniques that are already established for Green’s functions of linear BVPs.

Given the critical role that BVPs play in the mathematical analysis of constrained physical systems subjected to external forces, the DeepGreen architecture can be broadly applied in nearly every engineering discipline since BVPs are prevalent in diverse problem domains including fluid mechanics, electromagnetics, quantum mechanics, and elasticity. Importantly, DeepGreen provides a bridge between a classic and widely used solution technique to nonlinear BVP problems which generically do not have principled techniques for achieving solutions aside from brute-force computation. DeepGreen establishes this bridge by providing a transformation which allows linear superposition to hold. DeepGreen is a flexible, data-driven, deep learning approach to solving nonlinear boundary value problems (BVPs) using a dual-autoencoder architecture. The autoencoders discover an invertible coordinate transform that linearizes the nonlinear BVP and identifies both a linear operator *L* and Green’s function *G* which can be used to solve new nonlinear BVPs. We demonstrated that the method succeeds on a variety of nonlinear systems including nonlinear Helmholtz and Sturm–Liouville problems, nonlinear elasticity, and a 2D nonlinear Poisson equation. The method merges the strengths of the universal approximation capabilities of deep learning with the physics knowledge of Green’s functions to yield a flexible tool for identifying fundamental solutions to a variety of nonlinear systems.

Despite the success of the presented method and architecture, there are a few limitations that should be discussed regarding the architecture and the assumptions made in the design of the network. For example, the network assumes that the nonlinear system described by the data *can* be transformed from a nonlinear manifold to a linear manifold via the autoencoder architectures. It is possible that for some systems the transform does not exist, in which case we expect the architecture to approximate the manifold that linearizes the system. Experiments provided in the Supplementary Materials indicate that 100 experiments on the same system with the same data learned different transformations by the autoencoder. This indicates that the autoencoder learns different transforms from different initializations and implies that the learned transform is not unique. Additionally, the network can struggle with systems where the governing BVP does not have unique solutions. In this case, it is unclear how a training data set can be constructed which can appropriately guide the network to discovering an accurate transform. For example, consider a BVP that may have multiple or infinite solutions. The training data consists of vector pairs $\{{\mathbf {u}}_k,{\mathbf {F}}_k\}$. However, for any given ${\mathbf {F}}_k$ there are multiple solutions ${\mathbf {u}}_k$ which satisfy the BVP. Which one should be selected to use for training? These latter two limitations are the most important drawbacks of the current architecture: (i) the learned transform is not unique, and (ii) the architecture does not work on systems where multiple solutions can satisfy the BVP. For the BVPs considered here, the DeepGreen method was able to successfully generalize to be able to solve the BVPs for a cubic polynomial forcing function even though it had only been trained on cosine and Gaussian forcing functions. However, as stated by Mallat^54^, “Supervised learning is a high-dimensional interpolation problem.” Therefore, this type of generalization can only be achieved with a diverse enough set of training data. Additionally, the method cannot be expected to have accurate predictions for forcing functions with magnitude outside the range of magnitudes used for training.

## Methods: deep autoencoders for linearizing BVPs

Deep AEs have been used to linearize dynamical systems, which are initial value problems. We extend this idea to BVPs. To be precise, we consider BVPs of the form

11 $$\begin{aligned} N[u({\mathbf {x}})]&= F({\mathbf {x}}),&{\mathbf {x}}&\in \Omega , \end{aligned}$$

12 $$\begin{aligned} B[u({\mathbf {x}})]&= 0,&{\mathbf {x}}&\in \partial \Omega , \end{aligned}$$

where $\Omega$ is a simply connected open set in ${\mathbb {R}}^n$ with boundary $\partial \Omega$, *N* is a nonlinear differential operator, $F({\mathbf {x}})$ is the nonhomogeneous forcing function, *B* is a boundary condition, and $u({\mathbf {x}})$ is the solution to the BVP. We wish to find a pair of coordinate transformations of the form (5) and (6) such that *v* and *f* satisfy a linear BVP

13 $$\begin{aligned} L[v({\widehat{{\mathbf {x}}}})]&= f({\widehat{{\mathbf {x}}}}),&{\widehat{{\mathbf {x}}}}&\in {\widehat{\Omega }}, \end{aligned}$$

14 $$\begin{aligned} {\widehat{B}}[v({\widehat{{\mathbf {x}}}})]&= 0,&{\widehat{{\mathbf {x}}}}&\in \partial {\widehat{\Omega }}, \end{aligned}$$

where *L* is a linear differential operator and ${\widehat{{\mathbf {x}}}}$ is the spatial coordinate in the transformed domain ${\widehat{\Omega }}$ with boundary $\partial {\widehat{\Omega }}$. Although this work uses zero Dirichlet boundary conditions, there is nothing in the network design which prohibits use of the DeepGreen architecture with other types of boundary conditions. Because *L* is linear, there is a Green’s function $G({\widehat{{\mathbf {x}}}},{\xi })$ such that the solution *v* to the BVP (13) and (14) can be obtained through convolution of the Green’s function and transformed forcing function

15 $$\begin{aligned} v({\widehat{{\mathbf {x}}}}) = \int \limits _{{\widehat{\Omega }}} G({\xi },{\widehat{{\mathbf {x}}}}) f({\xi }) d{\xi }. \end{aligned}$$

The coordinate transformation along with the Green’s function of the linearized BVP provide the analog of a Green’s function for the nonlinear BVP (11) and (12). In particular, for a forcing function $F({\mathbf {x}})$, the transformed forcing function is $f = \varvec{\phi }(F)$. The solution to the linearized BVP can be obtained using the Green’s function $v = \int G({\xi },{\widehat{{\mathbf {x}}}}) f({\xi }) d {\xi }$. Then the solution to the nonlinear BVP (11) and (12) is obtained by inverting the coordinate transformation $u=\varvec{\psi }^{-1}(v)$ to obtain the solution to the nonlinear BVP, $u({\mathbf {x}})$.

The question that remains is how to discover the appropriate coordinate transformations $\varvec{\psi }$ and $\varvec{\phi }$. We leverage the universal approximation properties of neural networks in order to learn these transformations. In order to use neural networks, we first need to discretize the BVP. Let ${\mathbf {u}}$ be a spatial discretization of $u({\mathbf {x}})$ and ${\mathbf {F}}$ be a discretization of $F({\mathbf {x}})$. Then the discretized version of the BVP (11) and (12) is

16 $$\begin{aligned} {\mathbf {N}}[{\mathbf {u}}]&= {\mathbf {F}}, \end{aligned}$$

17 $$\begin{aligned} {\mathbf {B}}[{\mathbf {u}}]&= {\mathbf {0}}. \end{aligned}$$

Neural networks $\varvec{\psi }_u$ and $\varvec{\phi }_F$ are used to transform ${\mathbf {u}}$ and ${\mathbf {F}}$ to the latent space vectors ${\mathbf {v}}$ and ${\mathbf {f}}$

18 $$\begin{aligned} {\mathbf {v}}&= \varvec{\psi }_u({\mathbf {u}}), \end{aligned}$$

19 $$\begin{aligned} {\mathbf {f}}&= \varvec{\phi }_F({\mathbf {F}}), \end{aligned}$$

where ${\mathbf {v}}$ and ${\mathbf {f}}$ satisfy the linear equation

20 $$\begin{aligned} {\mathbf {L}}{\mathbf {v}}= {\mathbf {f}}, \end{aligned}$$

for some matrix ${\mathbf {L}}$, which is also learned. In order to learn invertible transforms $\varvec{\psi }_u$ and $\varvec{\phi }_F$, we construct the problem as a pair of autoencoder networks.

In this construction, the transforms $\varvec{\psi }_u$ and $\varvec{\phi }_F$ are the encoders and the transform inverses are the decoders. The network architecture and loss functions are shown in Fig. 2. The neural network is trained using numerous and diverse solutions to the nonlinear BVP (16) and (17), which can be obtained with many different forcings ${\mathbf {F}}_k$. Consider a dataset comprised of pairs of discretized solutions and forcing functions $\{{\mathbf {u}}_k,{\mathbf {F}}_k\}_{k=1}^N$. The loss function for training the network is the sum of six losses, each of which enforces a desired condition. The loss functions can be split into three categories:

1. *Autoencoder losses* We wish to learn invertible coordinate transformations given by Eqs. (18) and (19). In order to do so, we use two autoencoders. The autoencoder for ${\mathbf {u}}$ consists of an encoder $\varvec{\psi }_u$ which performs the transformation (18) and a decoder $\varvec{\psi }_u^{-1}$ which inverts the transformation. In order to enforce that the encoder and decoder are inverses, we use the autoencoder loss
  21 $$\begin{aligned} {\fancyscript{L}}_1 = \frac{1}{N} \sum _{k=1}^N \frac{\left\Vert {\mathbf {u}}_k - \varvec{\psi }_u^{-1} \circ \varvec{\psi }_u({\mathbf {u}}_k) \right\Vert _2^2}{\left\Vert {\mathbf {u}}_k\right\Vert _2^2}. \end{aligned}$$
  Similarly, there is an autoencoder for ${\mathbf {F}}$ where the encoder $\varvec{\phi }_F$ performs the transformation (19). This transformation also has an inverse enforced by the associated autoencoder loss function
  22 $$\begin{aligned} {\fancyscript{L}}_2 = \frac{1}{N} \sum _{k=1}^N \frac{\left\Vert {\mathbf {F}}_k - \varvec{\phi }_F^{-1} \circ \varvec{\phi }_F({\mathbf {F}}_k) \right\Vert _2^2}{\left\Vert {\mathbf {F}}_k\right\Vert _2^2}. \end{aligned}$$
2. *Linearity losses* In the transformed coordinate system, we wish for the BVP to be linear so that the operator can be represented by a matrix ${\mathbf {L}}$. The matrix ${\mathbf {L}}$ and the encoded vectors ${\mathbf {v}}$ and ${\mathbf {f}}$ should satisfy Eq. (20). This is enforced with the linear operator loss
  23 $$\begin{aligned} {\fancyscript{L}}_3 = \frac{1}{N} \sum _{k=1}^N \frac{\left\Vert {\mathbf {f}}_k - {\mathbf {L}}{\mathbf {v}}_k \right\Vert _2^2}{\left\Vert {\mathbf {f}}_k\right\Vert _2^2}. \end{aligned}$$
  The major advantage of working with a linear operator is that linear superposition holds. We use a linear superposition loss in order to further enforce the linearity of the operator in the latent space
  24 $$\begin{aligned} {\fancyscript{L}}_4 = \frac{1}{N^2} \sum _{j=1}^N \sum _{i=1}^N \frac{\left\Vert ({\mathbf {f}}_i+{\mathbf {f}}_j) - {\mathbf {L}}({\mathbf {v}}_i+{\mathbf {v}}_j) \right\Vert _2^2}{\left\Vert {\mathbf {f}}_i + {\mathbf {f}}_j \right\Vert _2^2}. \end{aligned}$$
3. *Cross-mapping losses* The losses described above are theoretically sufficient to find coordinate transformations for ${\mathbf {u}}$ and ${\mathbf {F}}$ as well as a linear operator ${\mathbf {L}}$. However, in practice the two autoencoders were not capable of generating the Green’s function solution. To rectify this, we add two “cross-mapping” loss functions that incorporate parts of both autoencoders. The first cross-mapping loss enforces the following mapping from ${\mathbf {u}}$ to ${\mathbf {F}}$. First, one of the solutions from the dataset ${\mathbf {u}}_k$ is encoded with $\varvec{\psi }_u$. This is an approximation for ${\mathbf {v}}_k$. This is then multiplied by the matrix ${\mathbf {L}}$, giving an approximation of ${\mathbf {f}}_k$. Then the result is decoded with $\varvec{\phi }_F^{-1}$. This gives an approximation of ${\mathbf {F}}_k$. The ${\mathbf {u}}$ to ${\mathbf {F}}$ cross-mapping loss is given by the formula
  25 $$\begin{aligned} {\fancyscript{L}}_5 = \frac{1}{N} \sum _{k=1}^N \frac{\left\Vert {\mathbf {F}}_k - \varvec{\phi }_F^{-1} \circ {\mathbf {L}}\circ \varvec{\psi }_u({\mathbf {u}}_k) \right\Vert _2^2}{\left\Vert {\mathbf {F}}_k\right\Vert _2^2}. \end{aligned}$$
  We can similarly define a cross-mapping from ${\mathbf {F}}$ to ${\mathbf {u}}$. For a forcing function ${\mathbf {F}}_k$ from the dataset, it is encoded with $\varvec{\phi }_F$, multiplied by the Green’s function (${\mathbf {G}}={\mathbf {L}}^{-1}$), and then decoded with $\varvec{\psi }_u^{-1}$ to give an approximation of ${\mathbf {u}}_k$. The ${\mathbf {F}}$ to ${\mathbf {u}}$ cross-mapping loss is
  26 $$\begin{aligned} {\fancyscript {L}}_6 = \frac{1}{N} \sum _{k=1}^N \frac{\left\Vert {\mathbf {u}}_k - \varvec{\psi }_u^{-1} \circ {\mathbf {L}}^{-1} \circ \varvec{\phi }_F({\mathbf {F}}_k) \right\Vert _2^2}{\left\Vert {\mathbf {u}}_k\right\Vert _2^2}. \end{aligned}$$

Note that this final loss function gives the best indication of the performance of the network to solve the nonlinear BVP (16) and (17) using the Green’s function. The strategy for solving (16) and (17) for a given discrete forcing function ${\mathbf {F}}$ is to encode the forcing function to obtain ${\mathbf {f}}= \varvec{\phi }_F({\mathbf {F}})$, apply the Green’s function as in Eq. (15) to obtain ${\mathbf {v}}$, and then decode this function to get the solution ${\mathbf {u}}= \varvec{\psi }_u^{-1}({\mathbf {v}})$. The discrete version of the convolution with the Green’s function given in Eq. (15) is multiplication by the matrix ${\mathbf {L}}^{-1}$.

For the encoders $\varvec{\phi }$ and $\varvec{\psi }$ and decoders $\varvec{\phi }^{-1}$ and $\varvec{\psi }^{-1}$, we use a residual neural network (ResNet) architecture^45^. The ResNet architecture has been successful in learning coordinate transformations for physical systems^15^. The use of ResNets is motivated by near-identity transformations in physics. We express each coordinate transformation as the sum of the identity transformation and a nonlinear residual transformation in the form of a neural network. The linear operator ${\mathbf {L}}$ is constrained to be a real symmetric matrix and therefore is self-adjoint. Additionally, ${\mathbf {L}}$ is initialized as the identity matrix. Therefore, ${\mathbf {L}}$ is strictly diagonally dominant for at least the early parts of training which guarantees ${\mathbf {L}}$ is invertible and well-conditioned. For more information on the network architecture and training procedure, see the Supplementary Materials.

**Figure 5:**
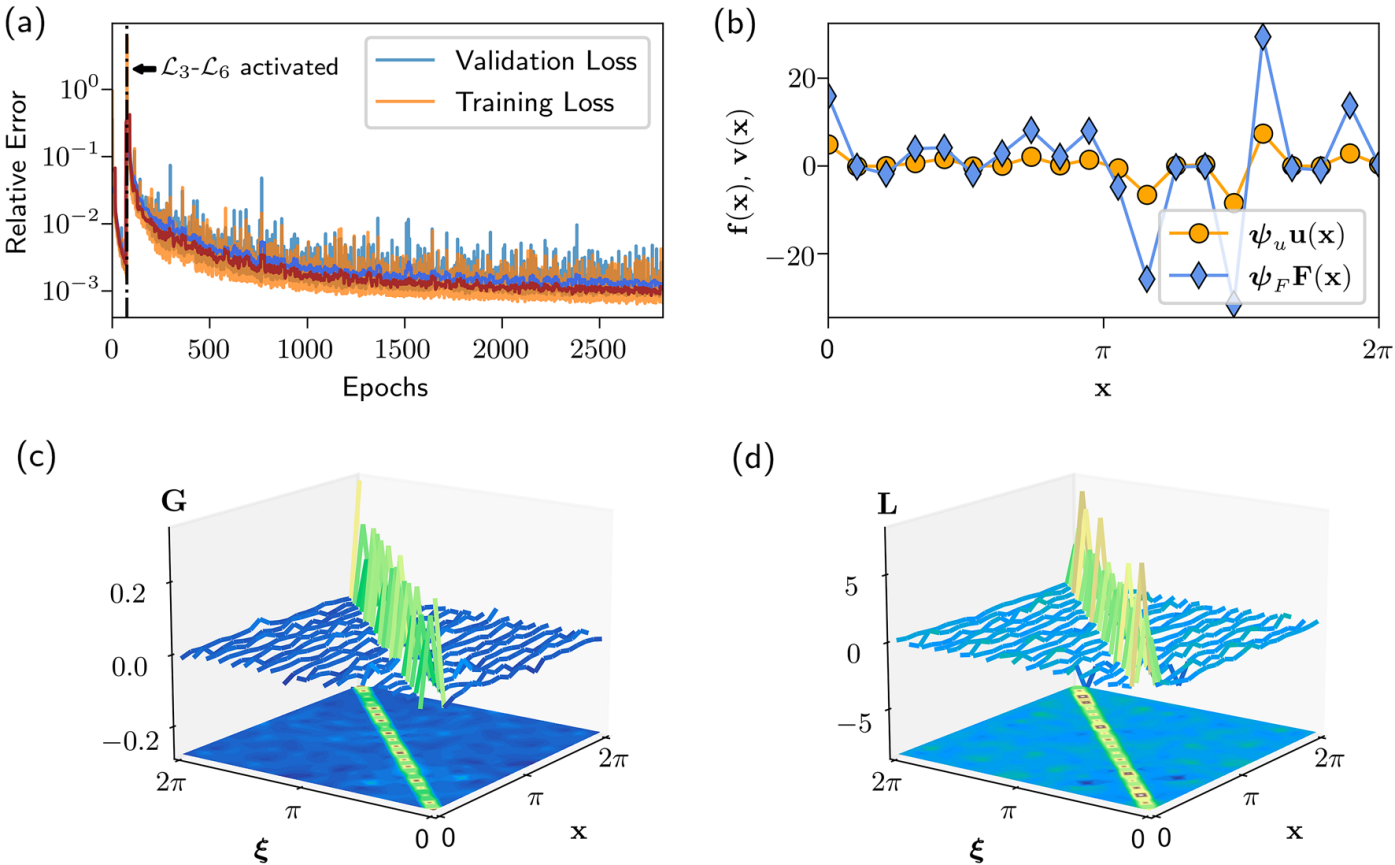
(**a**) Learning curve. This is a typical learning curve for the DeepGreen architecture. The vertical dashed line indicates where the training procedure transitions from autoencoders-only (only ${\fancyscript {L}}_1$ and ${\fancyscript {L}}_2$) to a full-network training procedure (all losses). (**b**) Latent space representations ${\mathbf {v}}_k$ and ${\mathbf {f}}_k$. The autoencoder transformation $\varvec{\psi }_u$ encodes ${\mathbf {u}}_k$ to the latent space, producing the vector ${\mathbf {v}}_k$ (orange). The forcing vector ${\mathbf {F}}_k$ is transformed by $\varvec{\psi }_F$ to the encoded vector ${\mathbf {f}}_k$ (blue). (**c,d**) Visualized operator and Green’s function. Discovered Green’s function ${\mathbf {G}}={\mathbf {L}}^{-1}$ and corresponding linear operator ${\mathbf {L}}$.

### Training the model: cubic Helmholtz

The architecture and methodology is best illustrated using the cubic Helmholtz equation as a basic example. The autoencoders used for the cubic Helmholtz equation are constructed with fully connected layers. In both autoencoders, a ResNet-like identity skip connection connects the input layer to the layer before dimension reduction in the encoder, and the first full-dimension layer in the decoder with the final output layer. The model is trained in a two-step procedure. First, the autoencoders are trained, without connection in the latent space, to condition the networks as autoencoders. In this first phase, only the autoencoder loss functions listed in Fig. 2 are active (${\fancyscript{L}}_1$ and ${\fancyscript{L}}_2$). After a set number of epochs, the latent spaces are connected by an invertible matrix operator, ${\mathbf{L}}$, and the remaining 4 loss functions in Fig. 2 become active (${\fancyscript{L}}_3$–${\fancyscript{L}}_6$). In the final phase of training, the autoencoder learns to encode a latent space representation of the system where properties associated with linear systems hold true, such as linear superposition.

Figure 5a shows a typical training loss curve. The vertical dashed line indicates the transition between the two training phases. The models in this work are trained for 75 epochs in the first autoencoder-only phase and 2750 epochs in the final phase. The first-phase epoch count was tuned empirically based on final model performance. The final phase epoch count was selected for practical reasons; the training curve tended to flatten around 2750 epochs in all of our tested systems.

The autoencoder latent spaces are critically important. The latent space is the transformed vector space where linear properties (e.g. superposition) are enforced which enables the solution of nonlinear problems. In the one-dimensional problems, the latent space vectors ${\mathbf {v}}$ and ${\mathbf {f}}$ are in ${\mathbb {R}}^{20}$. The latent spaces did not have any obvious physical interpretation, and qualitatively appeared similar to the representations shown in Fig. 5b. We trained 100 models to check the consistency in the learned model and latent space representations and discovered the latent spaces varied considerably (see the Supplementary Materials). This implies the existence of an infinity of solutions to the coordinate transform problem, which indicates further constraints could be placed on the model.

**Figure 6:**
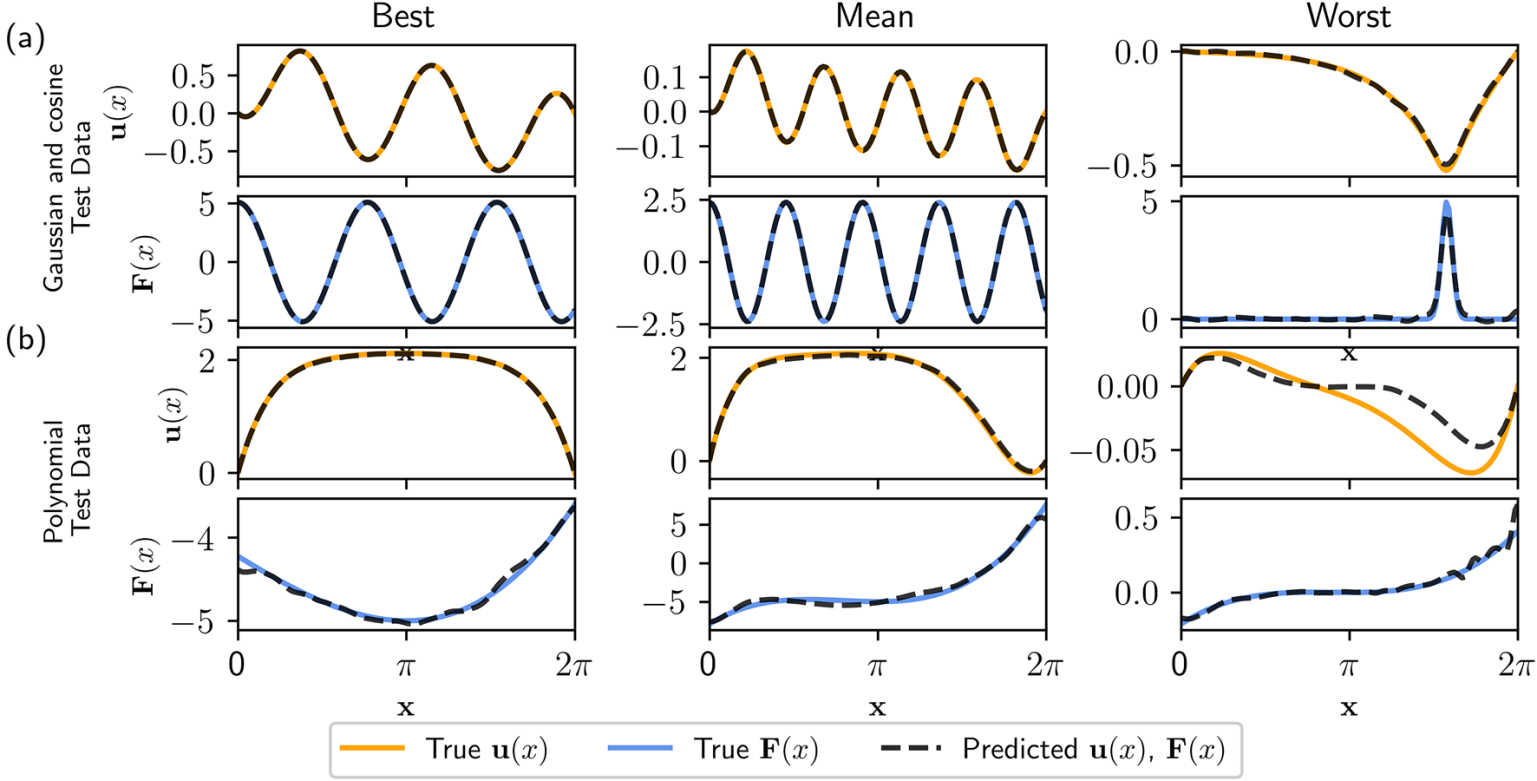
Model predictions on test data. The top row shows the true solution ${\mathbf {u}}_k(x)$ and the solution predicted by the network given the forcing ${\mathbf {F}}_k(x)$ using the Green’s function ${\mathbf {G}}$. The bottom row shows the true forcing function ${\mathbf {F}}_k(x)$ compared to the forcing computed by applying the operator ${\mathbf {L}}$ to the solution ${\mathbf {u}}_k$. Three columns show the best, mean, and worst case samples as evaluated by the sum of normalized $\ell 2$ reconstruction errors.

Despite lacking obvious physical interpretations, the latent space enables discovery of an invertible operator ${\mathbf {L}}$ which describes the linear system ${\mathbf {L}}[{\mathbf {v}}_k]={\mathbf {f}}_k$. The operator matrix ${\mathbf {L}}$ can be inverted to yield the matrix ${\mathbf {G}}$, where multiplication by ${\mathbf {G}}$ is the discrete version of convolution with the Green’s function. This allows computation of solutions to the linearized system ${\mathbf {v}}_k = {\mathbf {G}}[{\mathbf {f}}_k]$. An example of the operator ${\mathbf {L}}$ and its inverse ${\mathbf {G}}$ are shown in Fig. 5c,d. The operator and Green’s function shown in Fig. 5 display an important prominent feature seen in all of the results: a diagonally-dominant structure. We initialize the operator as an identity matrix, but the initialization had little impact on the diagonally-dominant form of the learned operator and Green’s function matrices (see the Supplementary Materials). The diagonally-dominant operators indicate that the deep learning network tends to discover a coordinate transform yielding a nearly-orthonormal basis, which mirrors the common approach of diagonalization in spectral theory for Hermitian operators. Furthermore, diagonally-dominant matrices guarantee favorable properties for this application such as being well-conditioned and non-singular.

We emphasize that training parameters and model construction choices used in this work were not extensively optimized. We expect the model performance can be improved in a myriad of ways including extending training times, optimizing model architecture, modifying the size of the latent spaces, restricting the form of the operator, and applying additional constraints to the model. The range of possibilities is combinatorially large and such an exploration is not the main scope of the present work; our focus is to illustrate the use of autoencoders as a coordinate transform for finding solutions to nonlinear BVPs.

**Figure 7:**
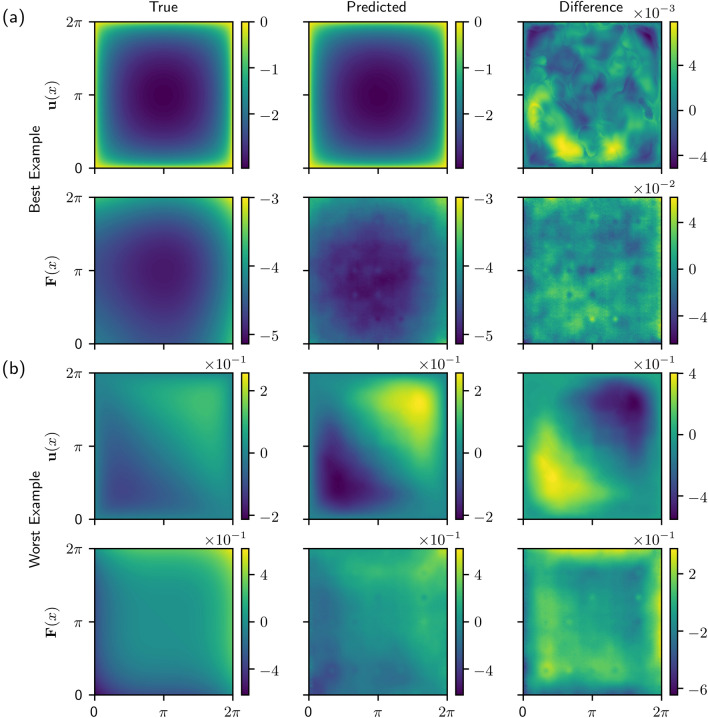
Model predictions for the (**a**) best and (**b**) worst examples from test data with cubic polynomial forcings. In both (**a,b**), the top row shows the true solution ${\mathbf {u}}({\mathbf {x}})$, the predicted solution using the Green’s function, and the difference between the true and predicted solution. The bottom row shows the true forcing function ${\mathbf {F}}({\mathbf {x}})$, the predicted forcing function, and the difference between the true and predicted forces. In order to account for the difference in scale between ${\mathbf {u}}({\mathbf {x}})$ and ${\mathbf {F}}({\mathbf {x}})$, the differences are scaled by the infinity norm of the true solution or forcing function ($\text {Difference} = (\text {True} - \text {Predicted})/ || \text {True}||_{\infty }$).

### Evaluating the model: cubic Helmholtz

The goal for this model is to find a Green’s function ${\mathbf {G}}$ for computing solutions ${\mathbf {u}}_k$ to a nonlinear BVP governed by (11) and (12) for a given forcing function ${\mathbf {F}}_k$. Similarly, we can estimate the forcing term, ${\mathbf {F}}_k$, given the solution ${\mathbf {u}}_k$. The model is consequently evaluated by its ability to use the learned Green’s function and operator for predicting solutions and forcings, respectively, for new problems from a withheld test data set.

Recall the original model is trained on data where the forcing function is a cosine or Gaussian function. As shown in Fig. 6a, the model performs well on withheld test data where the forcing functions are cosine or Gaussian functions, producing a cumulative loss around $10^{-4}$. The solutions ${\mathbf {u}}_k$ and forcing ${\mathbf {F}}_k$ are depicted for the best, mean, and worst samples scored by cumulative loss. It’s important to note the test data used in Fig. 6a is similar to the training and validation data. Because ML models typically work extremely well in interpolation problems, it is reasonable to expect the model to perform well on this test data set.

As an interesting test to demonstrate the ability of the model to extrapolate, we prepared a separate set of test data $\{{\mathbf {u}}_k,{\mathbf {F}}_k\}_{k=1}^N$ containing solutions where ${\mathbf {F}}_k$ are cubic polynomial forcing functions. Explicit formulas for the cubic polynomial forcing functions are found in the Supplementary Materials. This type of data was not present in training, and provides some insight into the generality of the learned linear operator and Green’s function matrices. Figure 6b shows examples of how the model performs on these cubic polynomial-type forcing functions. Similar to Fig. 6a, the best, mean, and worst samples are shown as graded by overall loss. Figure 6 provides some qualitative insight into the model’s performance on specific instances selected from the pool of evaluated data. A quantitative perspective of the model’s performance is shown in the summary boxplot provided in Fig. 3d. This box plot shows statistics (median value, $Q_1$, $Q_3$, and range) for the six loss functions evaluated on the similar (cosine and Gaussian) test data.

### Training the model: two-dimensional Poisson equation

The network architecture of the encoders and decoders for the two-dimensional example differs from the one-dimensional examples. Instead of fully connected layers, convolutional layers were used in the encoders and decoders. However, we still use a ResNet architecture. Additionally, the latent space vectors are in ${\mathbb {R}}^{200}$. Full details on the network architecture can be found in the Supplementary Materials. Note that the method proposed for discovering Green’s functions allows for any network architecture to be used for the encoders and decoders. For the one-dimensional example, similar results were obtained using fully connected and convolutional layers. However, the convolutional architecture was better in the two-layer case and also allowed for a more manageable number of parameters for the wider network that resulted from discretizing the two-dimensional space.

### Evaluating the model: two-dimensional Poisson equation

**Figure 8:**
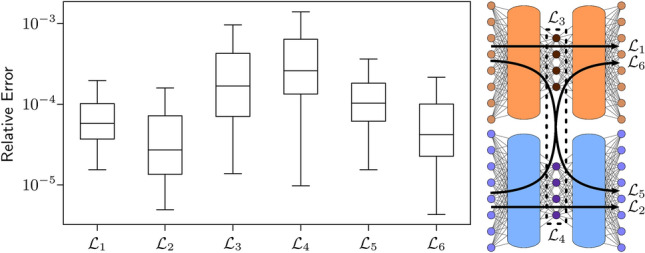
Two-dimensional Poisson model performance summary. Distribution of loss values are shown for every sample in the test data set. Model loss functions are minimized during training, making them a natural metric to use for summarizing performance.

The two-dimensional Poisson equation was also evaluated on test data that has cubic polynomial forcing functions, a type of forcing function not found in the training data. The best and worst examples are shown in Fig. 7. Although the model does not perform as well for test data which is not similar to the training data, the qualitative features of the predicted solutions are still consistent with the true solutions. Figure 8 shows a box plot of the model’s performance on the similar (cosine and Gaussian forcing) test data. The results are similar to the one-dimensional results, and, in fact, better than the biharmonic operator model.

## Supplementary Information

Supplementary Information.

## Data Availability

The code for this project is available on GitHub at https://github.com/sheadan/DeepGreen.
