# Supplementary material for: DeepGreen: deep learning of Green’s functions for nonlinear boundary value problems
Source: Sci Rep. 2021 Nov 3;11:21614. doi: 10.1038/s41598-021-00773-x (PMC8566504; doi:10.1038/s41598-021-00773-x)
Supplement: Supplementary file 1 — Supplementary Information. [file 41598_2021_773_MOESM1_ESM.pdf]

Supplementary Information for DeepGreen: Deep Learning of Green's Functions for Nonlinear Boundary Value Problems

Craig R. Gin[1,*,+], Daniel E. Shea[2,*,+], Steven L. Brunton[3], and J. Nathan Kutz[4]

[1]Department of Population Health and Pathobiology, North Carolina State University, Raleigh, NC 27695, United States
[2]Department of Materials Science and Engineering, University of Washington, Seattle, WA 98195, United States
[3]Department of Mechanical Engineering, University of Washington, Seattle, WA 98195, United States
[4]Department of Applied Mathematics, University of Washington, Seattle, WA 98195, United States
[*]crgin@ncsu.edu and sheadan@uw.edu
[+]these authors contributed equally to this work

Data Generation

1D Problems

The data for all of the one-dimensional systems are created using the same method and forcing functions. Each solution is computed on an evenly-spaced 128-point grid using MATLAB's bvp5c solver with a relative error tolerance of 10^{-8} and an absolute error tolerance of 10^{-10}. The forcing functions $\mathbf{F}_k(x)$ are designed to yield a variety of solutions $\mathbf{u}_k$ such that $||\mathbf{u}_k||_2 \simeq 1$.

The training data consists of two types of systems: Gaussian-forced and cosine-forced systems. The Gaussian-forced systems have forcing functions of the form

$$F_k(x) = a \exp\left(\frac{-(x-b)^2}{2c^2}\right),$$

where $a \in \{-25,-20,-15,-10,-5,5,10,15,20,25\}$, $b \in \{0, 2\pi/19, 4\pi/19, \ldots, 2\pi\}$, and $c \in \{0.1, 0.3, 0.5, \ldots, 4.9\}$. The cosine forcing functions are of the form

$$F_k(x) = \alpha \cos(\beta x),$$

where $\alpha \in \{1, 1.1, 1.2, \ldots, 10\}$ and $\beta \in \{1, 1.05, 1.10, \ldots, 5\}$. This gives a total of 5000 Gaussian-forced solutions and 7371 cosine-forced solutions. For the cubic Helmholtz equation and the nonlinear Sturm–Liouville equation with spatially-varying coefficients, all of the 12371 solutions were within the error tolerance. However, there were 97 solutions of the nonlinear biharmonic equation that did not meet the error tolerance and were therefore discarded. Of the remaining data, 10% are randomly chosen and withheld as test data, 80% are used as training data, and 20% are used as validation data.

In order to test the ability of the network to generalize, we also have another test data set that consists of solutions with cubic forcing functions of the form

$$F_i(x) = \gamma(x-\pi)^3,$$

where $\gamma \in \{0.01, 0.03, 0.05, \ldots, 0.29\}$, and cubic forcing functions of the form

$$F_i(x) = \gamma(x-\pi)^3 + \zeta(x-\pi)^2 + \psi,$$

where $\gamma \in \{0.01, 0.03, 0.05, \ldots, 0.29\}$, $\zeta \in \{0.01, 0.03, 0.05, \ldots, 0.49\}$, and $\psi \in \{-5, -4, -3, \ldots, 5\}$. There are a total of 4140 solutions with cubic forcing functions.

2D Problem

The two-dimensional data satisfies the nonlinear Poisson equation in the manuscript. The solutions are computed with a finite element method using the DOLFIN library[1] of the FEniCS Project[2,3]. The forcing functions are similar to the one-dimensional

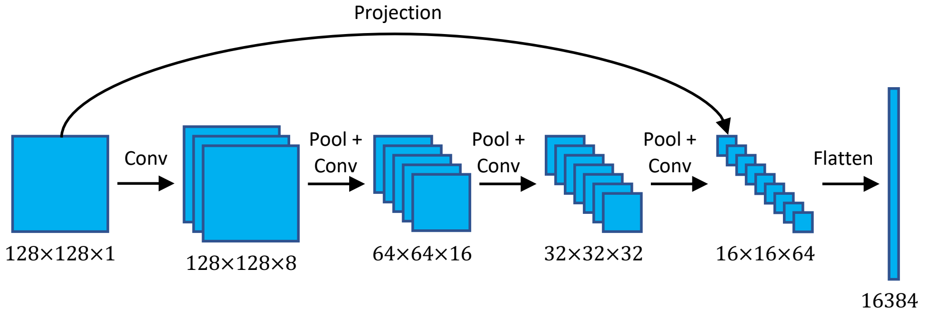

Supplementary Figure S1. Encoder network architecture for the two-dimensional data. All convolutional layers use 4×4 kernels with stride size 1, zero-padding, and ReLU activation functions. All pooling layers are average pooling layers with pool size 2 and stride size 2.

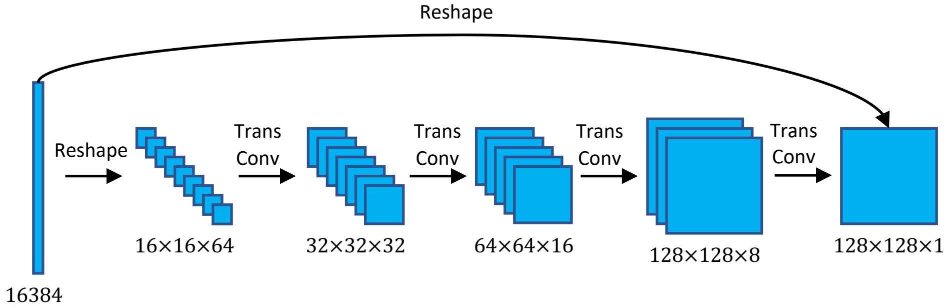

Supplementary Figure S2. Decoder network architecture for the two-dimensional data. All transposed convolutional layers use 4×4 kernels with stride size 2, zero-padding, and ReLU activation functions except for the last layer which has stride size 1.

data in that there are Gaussian and cosine forcing functions along with a separate data set of cubic polynomial forcing functions used to test the ability of the network to generalize. The Gaussian forcing functions are of the form

$$F_k(x,y) = a \exp\left(\frac{-(x-b_x)^2 - (y-b_y)^2}{2c^2} \right),$$

where $a \in \{-25,-20,-15,-10,-5,5,10,15,20,25\}$, $b_x, b_y \in \{\pi/3, 2\pi/3, \pi, 4\pi/3, 5\pi/3\}$, and $c \in \{0.1, 0.3, 0.5, \ldots, 4.9\}$. The cosine forcing functions are of the form

$$F_k(x,y) = \alpha \cos(\beta_x x)\cos(\beta_y y),$$

where $\alpha \in \{1, 1.1, 1.2, \ldots, 10\}$ and $\beta_x, \beta_y \in \{1, 1.5, 2, \ldots, 5\}$. There are cubic forcing functions of the form

$$F_i(x,y) = \gamma_x (x-\pi)^3 + \gamma_y (y-\pi)^3,$$

where $\gamma_x, \gamma_y \in \{0.01 + 0.28k/3 | k = 0,1,2,3\}$, and cubic forcing functions of the form

$$F_i(x,y) = \gamma_x (x-\pi)^3 + \gamma_y (y-\pi)^3 + \zeta_x (x-\pi)^2 + \zeta_y (y-\pi)^2 + \psi,$$

where $\gamma_x, \gamma_y \in \{0.01 + 0.28k/3 | k = 0,1,2,3\}$, $\zeta_x, \zeta_y \in \{0.01, 0.07, 0.13, 0.19, 0.25\}$, and $\psi \in \{-5,-4,-3,\ldots,5\}$. There are 6250 solutions with Gaussian forcing functions, 7371 solutions with cosine forcing functions, and 4416 solutions with cubic forcing functions.

Neural Network Implementation Details

The model training procedure is kept constant for all of the examples in this work. The networks are optimized with an Adam optimizer ($\beta_1 = 0.9$, $\beta_2 = 0.999$). Every numerical experiment starts by training a set of 20 models for a 'small' number of epochs. Each of the 20 models has a randomly selected learning rate for the Adam optimizer, uniformly selected between 10^{-2}

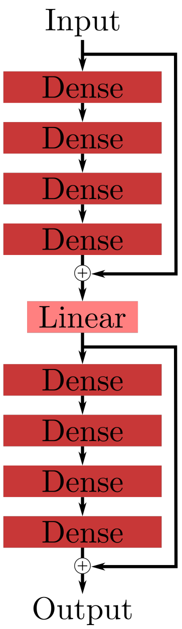

Supplementary Figure S3. Layer-by-layer autoencoder architecture for 1D problems.

and 10^{-5}. The initial training period consists of two phases: autoencoder-only (75 epochs) and full model (250 epochs). The autoencoder-only phase only enforces the autoencoder losses $\mathscr{L}_1$ and $\mathscr{L}_2$ during backpropagation. A checkpoint algorithm is used to keep track of the model with the lowest overall loss throughout the training procedure. At the end of the initial period, the best model is selected and the others are eliminated. The best model is trained for an additional 2500 epochs.

There are two network architectures in this work. The architectures depicted in Figs. S1 and S2 are applied to the two-dimensional nonlinear Poisson BVP. The architecture depicted in Fig. S3 is applied to one-dimensional problems.

The two architectures have a few training variables in common. Both models use variance scaling initialization, ℓ_2 regularization ($\lambda = 10^{-6}$), and ReLu activation functions for fully connected (1D architecture) and convolutional (2D architecture) layers. Notably, the two layers immediately before and after the latent space do not have activation functions. A normalized mean squared error loss function is used for all of the loss functions, as described in the manuscript. The models are trained in batches of 64 samples.

The 2D architecture utilizes convolutional layers and pooling layers, as shown in Figs. S1 and S2. All convolutional layers use a kernel size of 4×4. There are differences between the convolutional layers in the encoder and the convolutional layers in the decoder. The encoder convolutional layers use a stride size of 1×1 and an increasing number of filters $(8, 16, 32, 64)$. The deconvolutional layers use a stride size of 2×2 with decreasing filter size $(32, 16, 8)$. Pooling layers are similar for both the encoder and decoder with a stride size of 2×2 and a pool size of 2×2.

Additional Results

The repeatability of the results and models learned by the DeepGreen architecture are interesting to study from the perspective of operator convergence and latent space representations. In both cases, we aim to investigate the convergence of the model parameters to determine if the learned latent spaces and operators are unique or non-unique.

Operator Initialization

We repeat the training procedure for DeepGreen with three different initialization approaches for the operator L. Again, we train with data from the example nonlinear cubic Helmholtz model. This experiment focuses on comparing the initial values of the operator L with the final values of the operator at the end of training to determine if the DeepGreen approach tends to converge to a specific operator construction. The results in Fig. S4 show the initial and final operator for identity-initialized, randomly initialized, and Toeplitz-initialized operator matrices. Impressively, the result shows that the network tends to learn operators with diagonal dominance for all of the tested initialization strategies. This approach, which DeepGreen appears to prefer, draws strong parallels to the coordinate diagonalization approach commonly used in physics.

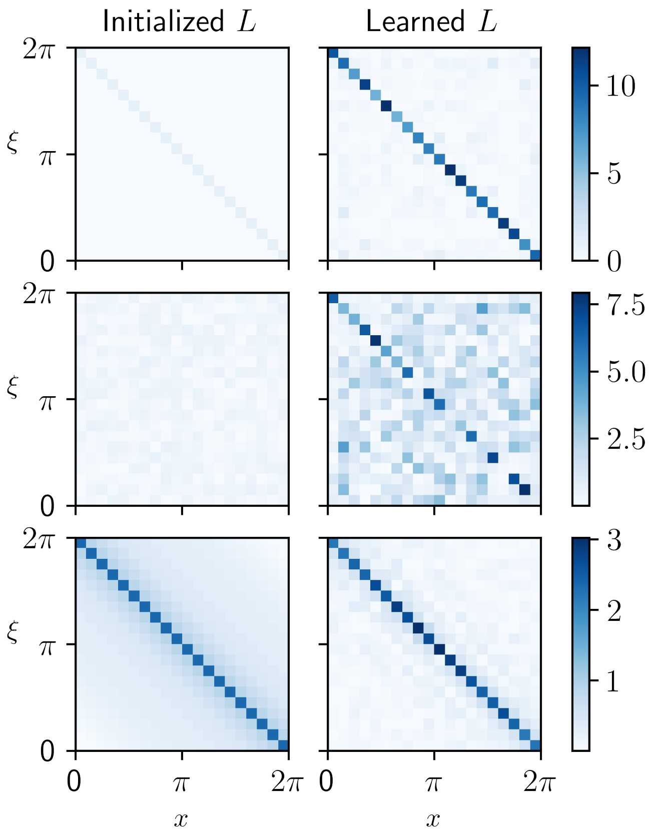

Supplementary Figure S4. Initial vs learned operators for an operator matrix L for different initial conditions. The top row shows identity matrix initialization, the middle row shows random initialization (He normal), and the bottom row shows a Toeplitz gradient initialization.

Latent Space Analysis

We repeat the training procedure for the example system, the nonlinear cubic Helmholtz model, a total of one hundred times. A single sample was selected from the training data and the latent space representation, $\mathbf{v}_i$ and $\mathbf{f}_i$, of the input vectors $\mathbf{u}_i$ and $\mathbf{F}_i$ are computed. Statistics for the latent space representations are presented in Fig. S5. It is evident that the latent space vectors are not identical between runs, and that the values in the vector do not follow any particular statistical distribution. This information implies that the learned weights in the model and the learned latent space representations vary for each training instance and do not appear to converge to a single representation.

Residual network architecture

All of the autoencoders used in this work use a residual network (ResNet) architecture. In order to demonstrate the advantage of the ResNet architecture, we trained six models using the DeepGreen architecture for each of the four systems. Three of the models use the ResNet skip connections, while three do not use the ResNet architecture.

For the two simplest systems, the nonlinear cubic Helmholtz equation and the nonlinear Sturm–Liouville equation, the difference between the models with and without the ResNet skip connections was negligible. For the nonlinear cubic Helmholtz equation, the mean validation loss for the non-ResNet models was 2.7×10^{-3} and the median validation loss was 2.4×10^{-3}. Using the ResNet architecture resulted in a mean validation loss of 3.5×10^{-3} and a median validation loss of 8.8×10^{-4}. The ResNet architecture resulted in a lower median validation loss but a higher mean due to one of the three models performing much more poorly than the other two. The results for the nonlinear Sturm–Liouville system are analogous. With a non-ResNet architecture, the mean validation loss was 4.5×10^{-3} and the median validation loss was 4.0×10^{-3}. With a ResNet architecture, the mean validation loss was 5.7×10^{-3} and the median validation loss was 3.1×10^{-3}. Therefore, the use of the ResNet architecture produced similar results to a non-ResNet architecture for these two simple systems.

For the two systems that had larger losses — the nonlinear biharmonic equation in 1D and the 2D nonlinear Poisson

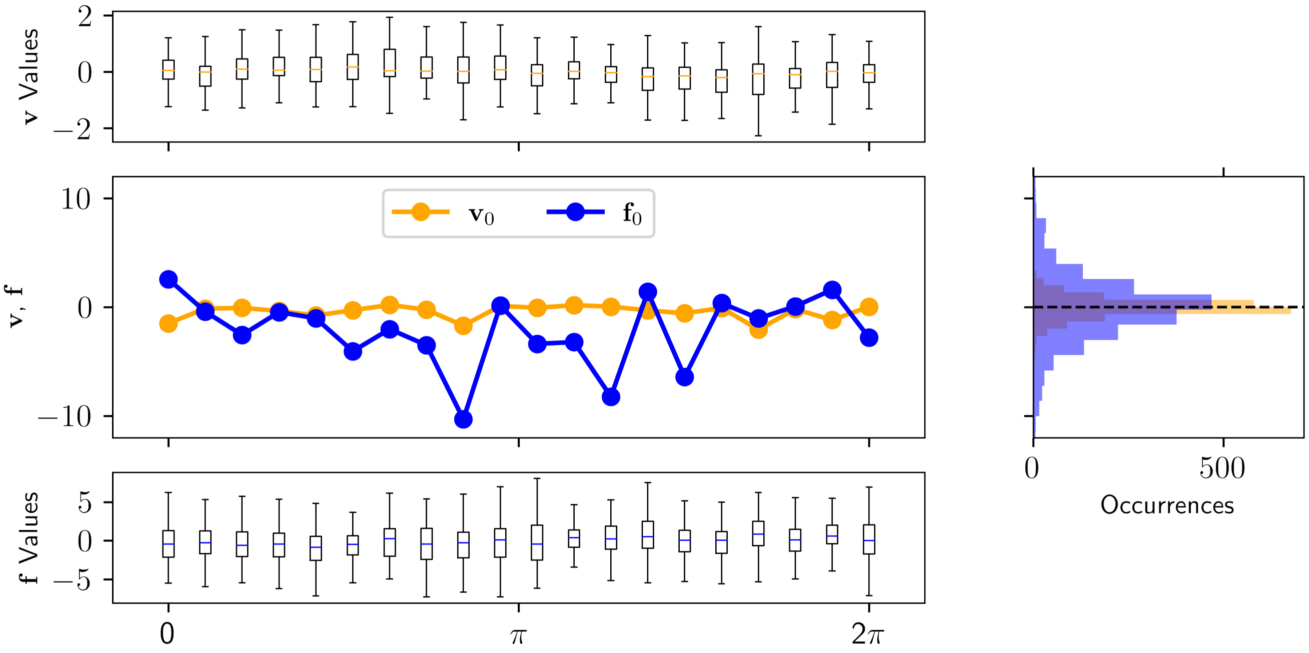

Supplementary Figure S5. Statistics of latent space values of a single sample over 100 experimental runs.

equation — the ResNet architecture was clearly superior to a non-ResNet architecture. For the nonlinear biharmonic equation, the ResNet architecture yields a mean validation loss of 2.5×10^{-2} and median validation loss of 2.8×10^{-2} for the three models, compared with 3.8×10^{-2} and 4.0×10^{-2}, respectively, for the non-ResNet architecture. Therefore, the ResNet architecture performed better in terms of both the mean and median loss. The ResNet architecture is absolutely vital for the nonlinear Poisson system. Without the ResNet architecture, the model essentially did not converge. Both the mean and median validation losses were 1.9×10^{0}. In contrast, the ResNet architecture had a mean validation loss of 1.8×10^{-2} and a median of 1.9×10^{-3}.

Comparison to baseline solvers

The DeepGreen architecture has several advantages over traditional BVP solvers with speed being one of the most striking. In order to demonstrate this, we performed speed tests of DeepGreen versus the numerical solvers that we used to generate the training and test data: MATLAB's bvp5c for the one-dimensional data and the finite element method of the DOLFIN library for the two-dimensional data. All speed tests were performed on a MacBook Pro with a 2.3 GHz Quad-Core Intel Core i7 processor and 32 GB of RAM. We recorded the time it took to solve the BVPs for each of the four model systems using the cubic polynomial forcing functions described above. The times in seconds are given in Supplementary Table S1. The last column gives the time using the traditional solver divided by the time using DeepGreen (labeled as "Speed up"). In all cases, the DeepGreen method is orders of magnitude faster than the traditional solver with the difference being most extreme for the one-dimensional systems.

One thing to note is that the times given for DeepGreen are conservative. As it is currently implemented, the predict method of the DeepGreen architecture takes a forcing function as input and outputs a prediction of the solution to the BVP, but it also takes a solution as input and outputs a prediction of the forcing function corresponding to that solution. Additionally, it runs both the forcing function and solution through their respective autoencoders. The times given in Supplementary Table S1 include all four of those evaluations and not just the time needed to produce a solution given a forcing function.

For several of the cubic polynomial forcing functions, MATLAB's bvp5c did not converge for the nonlinear biharmonic equation due to an initial guess that was outside the radius of convergence. This cannot happen for DeepGreen since no initial guess is required. In order to circumvent the issue of a poor initial guess, the BVP can be solved using a continuation method. However, this would require many nonlinear equation solves and the speed difference would be even more stark.

BVP	Traditional solver time (s)	DeepGreen time (s)	Speed up
cubic Helmholtz	5123.209476	0.101460	50494.87
nonlinear Sturm–Liouville	2866.761263	0.283519	10111.36
nonlinear biharmonic	5051.415544	0.287241	17585.98
nonlinear Poisson	3265.999239	19.462856	167.81

Supplementary Table S1. The time in seconds needed to solve the four different BVPs for the subic polynomial test data set. The traditional solver for the cubic Helmholtz, nonlinear Sturm–Liouville, and nonlinear biharmonic equations is MATLAB's bvp5c. The traditional solver for the nonlinear Poisson equation is a finite element method implemented by the DOLFIN library. The last column gives the time using the traditional solver divided by the time using DeepGreen.

References

1. Logg, A. & Wells, G. N. Dolfin: Automated finite element computing. *ACM Transactions on Math. Softw.* **37** (2010).

2. Alnæs, M. S. *et al.* The fenics project version 1.5. *Arch. Numer. Softw.* **3** (2015).

3. Logg, A., Mardal, K.-A., Wells, G. N. *et al. Automated Solution of Differential Equations by the Finite Element Method* (Springer, 2012).
